# Gender Differences and the Trend in the Acute Myocardial Infarction: A 10-Year Nationwide Population-Based Analysis

**DOI:** 10.1100/2012/184075

**Published:** 2012-09-10

**Authors:** Hung-Yu Yang, Jen-Hung Huang, Chien-Yeh Hsu, Yi-Jen Chen

**Affiliations:** ^1^Division of Cardiovascular Medicine, Department of Internal Medicine, Wan Fang Hospital, Taipei Medical University, No. 111, Section 3, Xinglong Road, Wenshan District, Taipei 116, Taiwan; ^2^Graduate Institute of Biomedical Informatics, College of Medicine, Taipei Medical University, Taipei, Taiwan; ^3^Graduate Institute of Clinical Medicine, College of Medicine, Taipei Medical University, Taipei, Taiwan

## Abstract

It is not clear whether gender is associated with different hospitalization cost and lengths for acute myocardial infarction (AMI). We identified patients hospitalized for primary diagnosis of AMI with (STEMI) or without (NSTEMI) ST elevation from 1999 to 2008 through a national database containing 1,000,000 subjects. As compared to that in 1999*~*2000, total (0.35‰  versus 0.06‰, *P* < 0.001) and male (0.59‰  versus 0.07‰, *P* < 0.001) STEMI hospitalization percentages were decreased in 2007*~*2008, but female STEMI hospitalization percentages were not different from 1999 to 2008. However, NSTEMI hospitalization percentages were similar over the 10-year period. The hospitalization age for AMI, STEMI, and NSTEMI was increased over the 10-year period by 14, 9, and 7 years in male, and by 18, 18, and 21 years in female. The female and male hospitalization cost and lengths were similar in the period. As compared to nonmedical center, the hospitalization cost for STEMI in medical center was higher in male patients, but not in female patients, and the hospitalization cost for NSTEMI was higher in both male and female gender. We found significant differences between male and female, medical center and non-medical center, or STEMI and NSTEMI on medical care over the 10-year period.

## 1. Introduction

Acute myocardial infarction (AMI) is one of the most important cardiovascular diseases with large medical expenditures. Decreased occurrences of AMI have been demonstrated epidemiologically [1, 2]. Through corrections of risk factors and intensive medical management, the mortality of AMI was significantly reduced [3–6]. 

Gender differences play an important role in the pathophysiology of AMI. Although coronary plaque rupture with acute thrombosis formation is common pathophysiology for men and women, women are usually older than men and associated with a low incidence of AMI, but with a higher mortality [7–16]. It has been proposed that gender differences on symptoms, awareness, prehospital delay, treatment responses, and complications may contribute to the different outcome. However, most epidemiological analyses were investigated in the Western communities. Moreover, most of the studies on AMI were evaluated in male gender. It is not clear whether gender differences also exist in the medical care of AMI in Asia. In addition, the gender effects on hospitalization cost between ST elevation (STEMI) and non-ST elevation (NSTEMI) AMI has not been evaluated. National Health Insurance (NHI) has provided medical care in all humans in Taiwan since 1995 [17]. Therefore, analyzing the database from NHI would provide the real-world community-based data on hospitalization cost and length of different gender. 

## 2. Method

### 2.1. Study Population

This study used the nationwide inpatient data from NHI, which can provide the database including the medical expenditure, admission periods, and co-morbidities [17, 18]. The NHI data included the data from the 23 million residents of the island's population, which contained 1,000,000 subjects from 1999 to 2008. The files were decoded by Graduate Institute of Biomedical Informatics, College of Medical Science and Technology, Taipei Medical University [19]. Patients with AMI were identified from the ICD-9 codes from 410.0 to 410.6 for STEMI and from 410.7 and 410.9 for NSTEMI [1]. We included the patients with the primary diagnosis with AMI (elevated and nonelevated) during hospitalization, which include the patients with any possibilities of co-morbidities without age limitation (age from 16 to 96 years old). We excluded the patients admitted more than one year, since the data on these patients cross over the next year and will not fit year analysis used in this study and excluded old MI patients admitted for other illness. The hospitalized percentages were calculated from the ratio of admitted patients with AMI over the total admitted patients. The medical centers and non-medical centers were qualified by Taiwan Joint Commission on Hospital Accreditation.

### 2.2. Statistical Analysis

Continuous variables were expressed as mean ± standard deviation (SD). Gender differences, medical center and non-medical center differences, and lower and higher hospitalization cost differences were compared by using unpaired Student's *t*-test, one-way analysis of variance (ANOVA), or two-way ANONA with post hoc of Fisher's method. Categorical variables were reported as frequencies and compared using a  *x* ^2^ or Fisher exact test if at least one cell had an expected cell count below 5. A two-tailed probability of *P* < 0.05 was considered statistically significant. All statistical analyses were performed with SPSS (version 13.0) or SigmaStat (version 3.5).

## 3. Results

**Figure 1:**
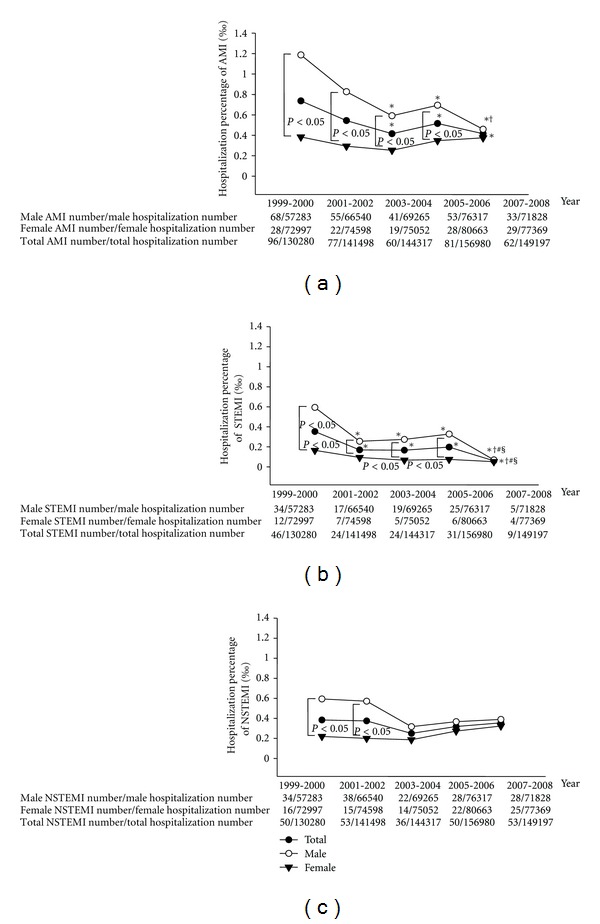
Hospitalization percentages of acute myocardial infarction (AMI, (a)), ST elevation MI (STEMI, (b)), or non-ST elevation MI (NSTEMI, (c)) from 1999 to 2008. *P < 0.05 versus 1999~2000, ^†^ *P* < 0.05 versus 2001~2002, ^#^ *P* < 0.05 versus 2003~2004, and ^§^ *P* < 0.05 versus 2005~2006.

Figure 1 shows hospitalization percentages of AMI from 1999 to 2008. The women composed a fewer percentage of AMI in this period, but the percentage increased progressively, whereas a similar gender percentage was noted in 2007~2008 (Table 1). As compared to that in 1999~2000, hospitalization percentages for total and male AMI were declined from 2003 to 2008, which was associated with an increase of total admission number and a decrease of total and male AMI admission number. However, in the female population, hospitalization percentages for total AMI were not changed. Hospitalization percentages of total and male STEMI were declined during the 10-year period with a decrease of total and male STEMI admission number. However, in the female population, hospitalization percentages of STEMI were not changed. Hospitalization percentages of STEMI were higher in male than in female from 1999 to 2006, but they were similar between male and female in 2007~2008. In contrast, hospitalization percentages of total, male, and female NSTEMI were not significantly changed in this period. Hospitalization percentages of NSTEMI were higher in male than in female from 1999 to 2002, but were similar between male and female in 2003~2008. 

**Figure 2:**
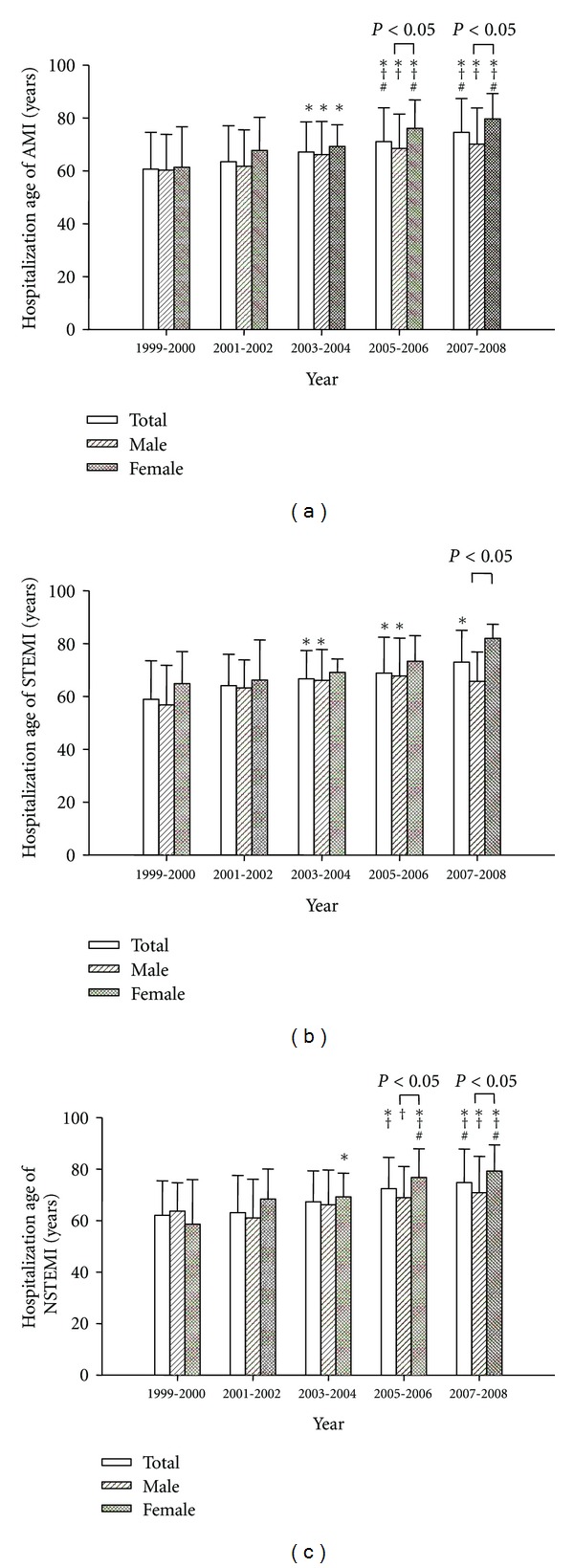
Hospitalization age of total, male and female acute myocardial infarction (AMI, (a)), ST elevation MI (STEMI, (b)), or non-ST elevation MI (NSTEMI, (c)) from 1999 to 2008. *P < 0.05 versus 1999~2000, ^†^ *P* < 0.05 versus 2001~2002, and ^#^ *P* < 0.05 versus 2003~2004. Only the patients with first onset AMI within the year were included.

As shown in Figure 2, the patient age of total, male, and female AMI was significantly increased over the 10-year period by 14, 10, and 19 years. Similarly, the patient age of total, male, and female STEMI was significantly increased over the 10-year period by 14, 9, and 18 years. The patient age of total, male, and female NSTEMI was significantly increased over the 10-year period by 14, 8, and 22 years. The hospitalization age of female AMI and NSTEMI patients was older than males from 2005 to 2008, but the hospitalization age of female STEMI patients was insignificantly (*P* = 0.061) older than male patients in 2007~2008. Table 1 shows the co-morbidity in the AMI patients in this period. The incidences of hypertension, diabetes, dyslipidemia, heart failure, stroke, and chronic lung disease were similar in this period. 

**Figure 3:**
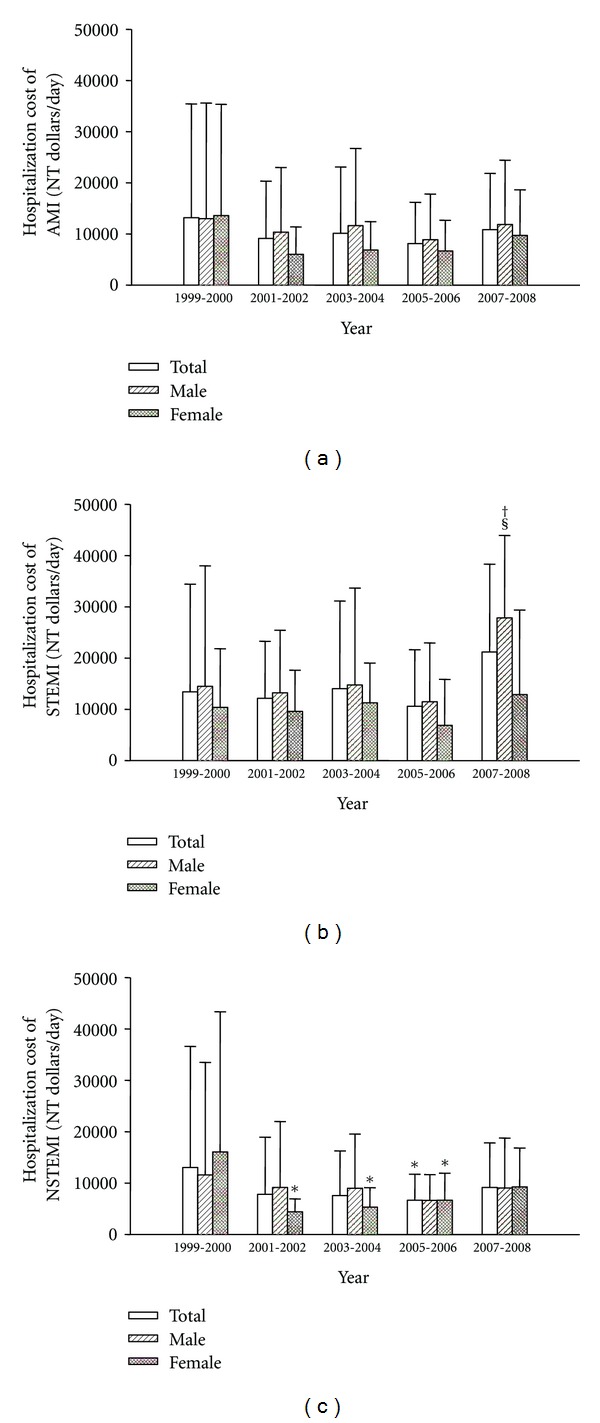
Hospitalization cost of total, male, and female acute myocardial infarction (AMI, (a)), ST elevation MI (STEMI, (b)), or non-ST elevation MI (NSTEMI, (c)) from 1999 to 2008. *P < 0.05 versus 1999~2000, ^†^ *P* < 0.05 versus 2001~2002, and ^§^ *P* < 0.05 versus 2005~2006.

We compared the average hospitalization cost over the 10-year period and found that the hospitalization cost of total, male, and female AMI was not significantly changed over the 10-year period (Figure 3). However, male STEMI has a higher hospitalization cost in 2007~2008 than in 2001~2002 and 2005~2006. 

**Figure 4:**
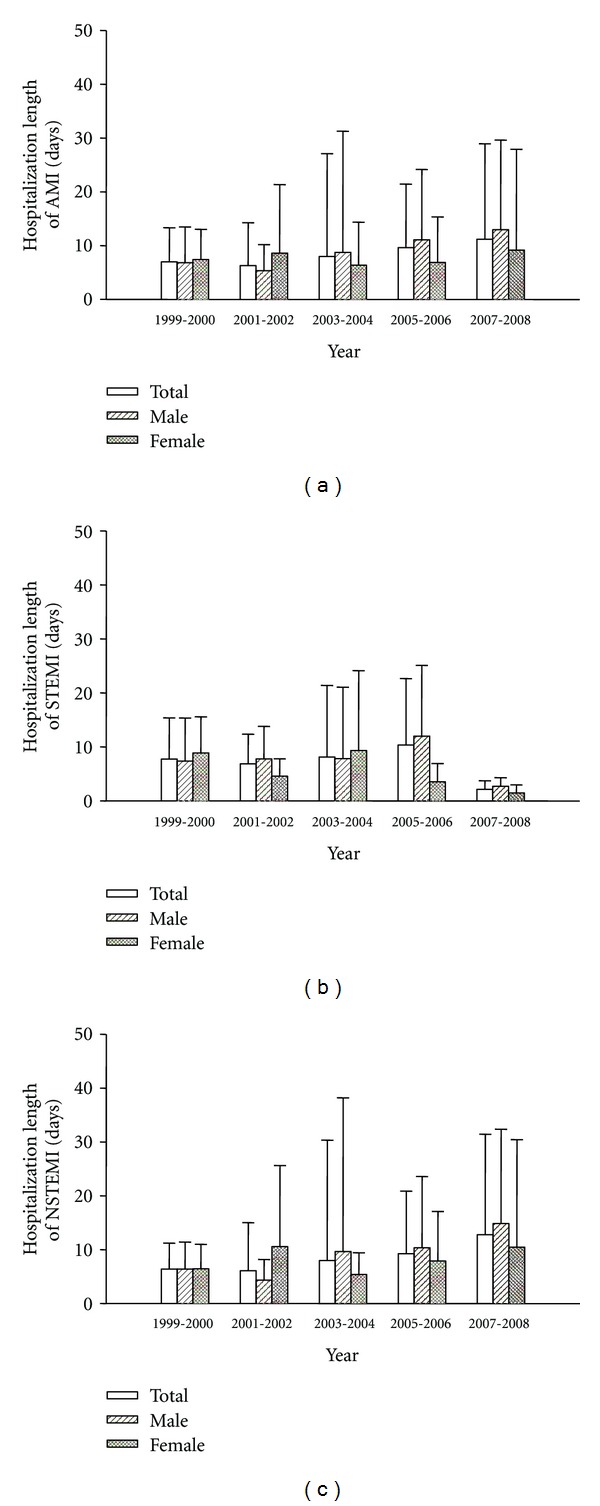
Hospitalization lengths of total, male, and female acute myocardial infarction (AMI, (a)), ST elevation MI (STEMI, (b)), or non-ST elevation MI (NSTEMI, (c)) from 1999 to 2008.

Figure 4 shows the comparisons of average hospitalization lengths over the 10-year period. The hospitalization lengths of total and female AMI were not significantly changed over the 10-year period, but were increased in male AMI in 2005~2006 and 2007~2008. The hospitalization lengths of total, male, and female STEMI were not significantly changed over the 10-year period. The hospitalization lengths of total and female NSTEMI were not significantly changed over the 10 year period, but were increased in male NSTEMI in 2005~2006 and 2007~2008. The hospitalization lengths of AMI, STEMI, and NSTEMI were similar between male and female over the 10-year period. 

Table 2 shows the average hospitalization age, hospitalization cost, and hospitalization lengths of AMI between medical centers and non-medical centers. As compared to non-medical centers, the hospitalization cost of total, female, and male AMI was larger in medial center. The hospitalization lengths of total and male AMI were longer in medical center, but the hospitalization lengths of female AMI were similar between medical center and non-medical center. In STEMI, the hospitalization cost of total and male patients was larger in medial center than in non-medical center, but was not significantly different in female patients (*P* = 0.07). The hospitalization lengths of total STEMI were longer in medical center than in non-medical center, but were not significantly different in male (*P* = 0.06) or female (*P* = 0.11) patients. In NSTEMI, the hospitalization cost of total, male, and female patients was larger in medical center than in non-medical center. The hospitalization lengths of total (*P* = 0.09), male (*P* = 0.18), and female (*P* = 0.29) patients were similar between medical center and non-medical center. The hospitalization age of AMI and STEMI was similar between center and non-center both in male and female gender. However, the hospitalization age of NSTEMI was younger in medical center than in non-medical center. 

**Figure 5:**
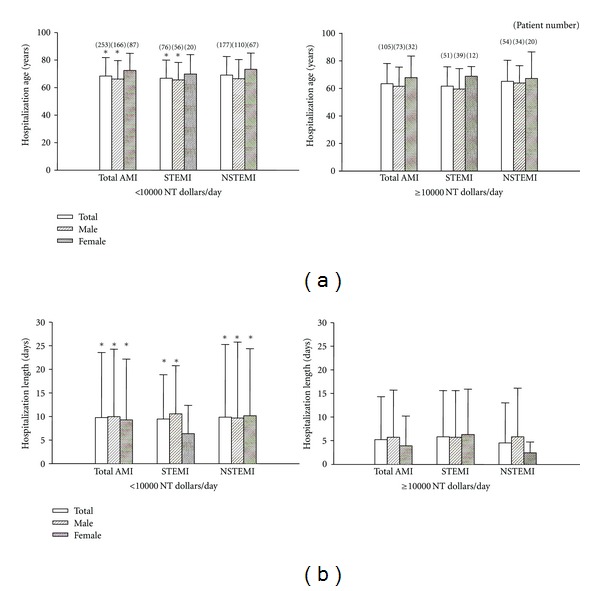
Hospitalization age and lengths in higher (≥10000 NT dollar/day) and lower cost AMI patients from 1999 to 2008. Panel (a) shows the average age from higher and lower AMI hospitalization cost. Panel (b) shows the average hospitalization lengths from higher and lower AMI hospitalization cost. *P < 0.05 versus higher cost patients (≥10000 NT dollars/day). Only the patients with first onset AMI within the year were included.

**Figure 6:**
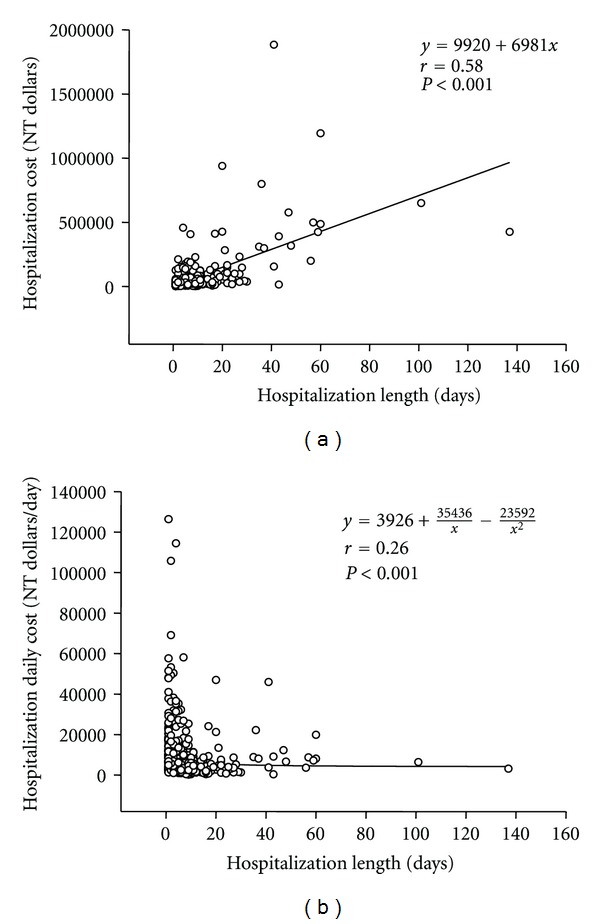
The correlation of hospital length and total cost or daily cost in AMI patients. The hospital length was linearly correlated with total cost (a) and inversely correlated with daily cost (b).

Because the average hospitalization cost from 1999–2008 is close to NT 10670, therefore, we choose this value (10000 NT dollars) for grouping.  Figure 5 showed the comparisons between the patients with higher (≥10000 NT dollar/day) or lower (<10000 NT dollar/day) hospitalization cost. The higher hospitalization cost in total and male patients with AMI and STEMI was younger and admitted shorter than the lower hospitalization cost total and male patients with AMI and STEMI, respectively. However, the age in higher and lower hospitalization cost was similar in female patients with AMI and STEMI and similar in total, male, and female NSTEMI patients. The hospitalization lengths were shorter in higher hospitalization cost patients than in lower hospitalization cost patients in female AMI and NSTEMI or male AMI, STEMI, and NSTEMI. Moreover, as shown in Figure 6, there was a linear correlation between the hospitalization length and the total cost for AMI and an inverse correlation between the hospitalization length and daily cost.

## 4. Discussion

In this study, through the reliable nationwide population-based dataset, which almost covers entire population in Taiwan, we demonstrated that the hospitalization percentages of AMI and STEMI were declined from 1999 to 2008. In this period, the total admission number was increased, but the number of total AMI or STEMI admission was decreased. Therefore, both factors contribute to the changes of AMI and STEMI admission percentage. The improvement would be caused by more aggressive risk factors control from medical systems and education on AMI prevention from the community and individual [1, 2]. However, this trend was more significant in male gender, which results in the near similar hospitalization percentages of AMI and STEMI between male and female in 2007~2008. This result may be caused by the gender differences because the hospitalization percentages of AMI and STEMI were lower in female than in male. Moreover, typical symptoms of coronary artery diseases or more attention and aggressive prevention in male gender may potentially result in the decline in MI or STEMI. In contrast, NSTEMI was not significantly changed both in male and female, which indicated different pathophysiology between STEMI and NSTEMI. 

In this study, the patient age of MI with or without ST elevation was significantly increased over the 10-year period. Similar to those in the previous studies, the patient age was older in female than in male, which implies the rather normal coronary artery and healthy life pattern in female than in male with the similar age. The decrease of AMI percentage of hospitalization and patient ages indicates that the improvement in treating coronary artery disease during this period. In contrast, the age of MI was reported to be similar within the 10-year period in USA [1].

In this study, for the first time, we evaluated the hospitalization cost of AMI over the 10-year period. Surprisingly, similar hospitalization cost over the 10-year period for treating MI was observed. Since the budget for health insurance in Taiwan was increased and mild inflation was demonstrated in this period, the similar hospitalization cost for treating MI actually means that the medical fare for MI treatment was decreased in this period. Although the reasons were not clear, cost restrictions from Bureau of NHI in Taiwan may play a role in this finding. In addition, the hospitalization cost may be underestimated since the expenses might not be coved by NHI and will not appear in the data. Additionally, this study also found a trend that the male patients had higher hospitalization cost for MI and STEMI. However, the hospitalization length seems to decrease in 2007~2008. In addition, we found an inverse correlation between the hospitalization length and daily cost. These findings suggest that more aggressive treatment of AMI may shorten the hospitalization length or more examinations and treatment were applied during the initial admission period.

 Medical centers had better facilities and personal supports for medical care with a large patients and procedure volume. It has been shown that volume of medical care will correlate with the outcome in AMI patients [20, 21]. However, it is not clear whether the hospitalization cost and lengths for AMI were different between the medical center and non-medical center. Over the 10-year period, the hospitalization cost of STEMI was larger in medial center than in non-medical center for male patients, but not in female patients. In contrast, the hospitalization cost of NSTEMI was larger in medial center than in non-medical center both for male and female patients. This finding may be due to that the severity of STEMI is different between medical center and non-medical center. However, the reimbursement for some of the medical services in medical center and non-medical center is different in Taiwan, which may also contribute to the differences medical cost between medical center and non-medical center. It is not clear whether the severity of AMI was different between the medical center and non-medical center. But we know in Taiwan the patient can be transferred to medical center if the severity increased. The longer hospitalization lengths in medical center indicate more severe and complicated AMI in the centers. We found that the patient age of AMI and STEMI was similar between medical center and non-medical center, but the patient age of NSTEMI was younger in medical center than in non-medical center. However, the underlying mechanisms are not clear in this study. 

We compared the differences between higher and lower hospitalization cost over the 10-year period and found that higher hospitalization cost patients were younger with shorter hospitalization lengths, which indicates that more aggressive treatment was used for these patients. However, this finding was mainly found in the male patients with STEMI.

Our findings should be interpreted with caution due to the study's limitations. First, we cannot identify whether the patients first admitted to non-medical center and then transferred to medical centers. Second, the results in this study are from the nationwide database, therefore the detail medications and management for these patients were not clear. In addition, the patient's biochemistry, image, and disease severity were not available. Moreover, although these analysis methods were widely used, we cannot exclude the possibility that ICD-9 codes might be incorrectly cited [1], which might influence the number of STEMI and NSTEMI analyzed in this study. In addition, co-morbidity may be underestimated in these patients because only 4 sets of secondary diagnosis were allowed in the NHI database. 

In conclusion, through the NHI database, we found an improvement of medical care for AMI. There were significant differences between male and female, center and non-medical center, or STEMI and NSTEMI over the 10-year period. 

## Figures and Tables

**Table 1:** Comorbidities in acute myocardial infarction patients from 1999 to 2008.

Variables	1999-2000	2001-2002	2003-2004	2005-2006	2007-2008	*P* value
No. of patients	96	77	60	81	62	0.001
Age (years)	61 ± 14	64 ± 14	68 ± 11	71 ± 13	75 ± 13	<0.001
Male number (percentage)	68 (71%)	55 (71%)	41 (68%)	53 (65%)	33 (53%)	0.154
STEMI	46 (48%)	24 (31%)	24 (39%)	31 (38%)	9 (15%)	<0.001
Male number in STEMI (percentage)	34 (74%)	17 (71%)	19 (79%)	25 (81%)	5 (56%)	0.596
Male number in NSTEMI (percentage)	34 (68%)	38 (72%)	22 (61%)	28 (56%)	28 (53%)	0.236
Coexisting conditions (ICD-9 code)						
Hypertension (401–405)	31 (32%)	26 (34%)	24 (40%)	19 (23%)	17 (27%)	0.274
Diabetes mellitus (250)	16 (17%)	16 (21%)	18 (30%)	15 (19%)	14 (23%)	0.353
Dyslipidemia (272)	10 (10%)	7 (9%)	7 (12%)	4 (5%)	4 (6%)	0.572
Other medical history-%						
Stroke or transient ischemic attack (433–435)	0 (0%)	1 (1%)	2 (2%)	3 (4%)	0 (0%)	0.195
Chronic heart failure (428)	5 (5%)	5 (6%)	8 (13%)	9 (11%)	12 (19%)	0.041
Chronic lung disease (490–496, 500–508)	9 (9%)	8 (10%)	4 (7%)	16 (20%)	7 (11%)	0.102
Neoplasms (140–239)	2 (2%)	0 (0%)	2 (3%)	1 (1%)	4 (6%)	0.136
Chronic kidney disease (585)	0 (0%)	4 (5%)	1 (2%)	2 (2%)	3 (5%)	0.206

STEMI: ST elevation MI, NSTEMI: non-ST elevation MI. Only the patients with first onset AMI within the year were included.

**Table 2:** Differences between medical center and non-medical center.

Total number (*n*)	AMI			STEMI			NSTEMI		
	Center (33)	Noncenter (343)	*P* value	Center (13)	Noncenter (121)	*P* value	Center (20)	Noncenter (222)	*P* value
Male	21 (64%)	229 (67%)	0.865	9 (69%)	91 (75%)	0.892	12 (60%)	138 (62%)	0.960
Total AMI age	65 ± 11	67 ± 14	0.264	63 ± 11	65 ± 14	0.603	66 ± 10	69 ± 14	0.341
Male	64 ± 11	65 ± 14	0.832	61 ± 13	65 ± 14	0.349	67 ± 10	66 ± 14	0.795
Female	65 ± 10	72 ± 14	0.072	68 ± 7	70 ± 13	0.792	63 ± 12	73 ± 14	0.044
Average cost/day (1000 NT dollars)	26 ± 32	9 ± 11	<0.001	26 ± 32	12 ± 13	0.003	25 ± 33	8 ± 9	<0.001
Male	26 ± 35	10 ± 12	<0.001	30 ± 38	13 ± 14	0.008	23 ± 33	8 ± 10	<0.001
Female	26 ± 29	7 ± 7	<0.001	19 ± 11	9 ± 10	0.070	29 ± 35	7 ± 6	<0.001
Average hospitalization days	14 ± 16	8 ± 12	0.011	14 ± 17	8 ± 8	0.022	14 ± 17	8 ± 14	0.092
Male	15 ± 16	8 ± 13	0.036	15 ± 19	8 ± 9	0.060	15 ± 14	8 ± 15	0.180
Female	13 ± 18	8 ± 11	0.149	12 ± 16	6 ± 5	0.114	13 ± 19	8 ± 12	0.287
Hypertension	9 (28%)	100 (31%)	0.765	3 (25%)	39 (34%)	0.532	6 (30%)	61 (28%)	0.918
Diabetes mellitus	5 (16%)	69 (21%)	0.460	2 (17%)	29 (25%)	0.512	3 (15%)	40 (19%)	0.664
Dyslipidemia	7 (22%)	23 (7%)	0.004	2 (17%)	10 (9%)	0.369	5 (25%)	13 (6%)	0.003
Stroke or transient ischemic attack	1 (3%)	5 (2%)	0.503	0 (0%)	2 (2%)	0.645	1 (5%)	3 (1%)	0.241
Chronic heart failure	3 (9%)	34 (10%)	0.852	1 (8%)	9 (8%)	0.950	2 (10%)	25 (12%)	0.806
Chronic lung disease	1 (3%)	40 (12%)	0.121	1 (8%)	15 (13%)	0.640	0 (0%)	25 (12%)	0.103
Neoplasms	1 (3%)	8 (2%)	0.817	1 (8%)	1 (1%)	0.048	0 (0%)	7 (3%)	0.408
Chronic kidney disease	2 (6%)	8 (2%)	0.214	1 (8%)	2 (2%)	0.152	1 (5%)	6 (3%)	0.591

AMI: acute myocardial infarction, STEMI: ST elevation MI, and NSTEMI: non-ST elevation MI. Only the patients with first onset AMI within the year were included.
